# Effect of Immunosuppression on Newcastle Disease Virus Persistence in Ducks with Different Immune Status

**DOI:** 10.5402/2012/253809

**Published:** 2012-01-23

**Authors:** Lucy W. Njagi, Phillip N. Nyaga, Lilly C. Bebora, Paul G. Mbuthia, Uswege M. Minga

**Affiliations:** ^1^Department of Veterinary Pathology, Microbiology and Parasitology, University of Nairobi, P.O. Box 29053-00625, Kangemi, Kenya; ^2^Department of Life Sciences, FSTES, African Council for Distance Education—Technical Committee on Collaboration (ACDE-TCC), Open University of Tanzania, P.O. Box 23409, Dar es Salaam, Tanzania

## Abstract

This study was carried out to verify the possibility that ducks are sources of Newcastle disease (ND) virus infection for chickens in mixed flocks. Immunosuppressed (IS) and non immunosuppressed (NIS) birds, at three different antibody levels (medium, low and absent) were used; the titres having been induced through vaccination, and Immunosuppression done using dexamethazone. Each of the 3 respective groups was further divided into 2 groups of about 12 ducks each: one challenged with velogenic ND virus; the other not challenged. Selected ducks from all groups had their antibody titres monitored serially using hemagglutination inhibition test, while two birds from each of the challenged groups were killed and respective tissues processed for ND viral recovery, using chicken embryo fibroblasts. In general, antibody titres of IS and NIS challenged ducks were significantly higher than their unchallenged counterparts (*P* < 0.05). Non-challenged pre-immunised ducks had a progressive decrease in antibody levels; non-immunised ducks did not seroconvert. Newcastle disease virus was isolated from livers and kidneys of the challenged ducks throughout the experimental period; indicating a possibility of viral excretion, especially when the birds are stressed. It, therefore, provides another possible model of viral circulation within mixed flocks.

## 1. Introduction

Village indigenous birds are constantly exposed to immunosuppressive conditions such as aflatoxicosis and infectious bursal disease virus [1]. In addition, management and ecological factors such as confinement, climatic and seasonal fluctuations, poor feeding, and worm infestations have been associated with stress and reduced immune response [2]. Stressful factors have been reported to cause functional and morphological changes in chickens [3]. 

In Tanzania, it was observed that Newcastle disease (ND) was a greater problem in villages with ducks [4]. Earlier reports indicated that Newcastle disease virus (NDV) persisted for a long time in a flock of ducks in a village situation in Indonesia [5]. However, the factors leading to shedding of the virus by the carrier ducks are not well documented. 

It was hypothesized that immunosuppression of immunised carrier ducks does not influence persistence of NDV in these birds. In this experiment, dexamethasone was used to simulate stress in village indigenous ducks. Thus, the aim of the present study was to determine the effect of immunosuppression on the viral persistence and immune status of ducks. It was designed to simulate field situation where ducks that have varying levels of NDV antibodies undergo immunosuppression in the presence of high NDV challenge.

## 2. Materials and Methods

### 2.1. Experimental Birds

One-day-old indigenous ducklings were hatched from the duck flock maintained at the University of Nairobi premises. All the birds were reared in isolation and transferred to experimental units at one year of age. They were wing tagged, tested, and confirmed to be free of NDV and respective antibodies. Water and food were provided *ad libitum.*

### 2.2. Inactivated Vaccine and Viral Inoculum

A Kenyan virulent Newcastle Disease virus isolate (vNDV) was obtained from the repository maintained at the University of Nairobi and characterized by standard methods [6]. This isolate was used to prepare an inactivated vaccine to immunise the ducks. The inactivated vaccine was prepared by mixing 40% formalin and allantoic fluid with a titer of 2^9^ of vNDV in a ratio of 1 : 40, that is, formalin to virus [6]. The reparation was kept at room temperature (24°C to 26°C) for 24 hours before use. The inactivation of the virus was confirmed through inoculation of embryonated eggs. All the ducks were vaccinated via an initial dose of 1 mL of the vaccine intramuscularly on the thighs and a booster of 0.5 mL of the same vaccine 16 days later. The live virulent Kenyan Newcastle diseases virus isolate, previously characterized by standard methods [6], was later used to challenge vaccinated and naive ducks.

### 2.3. Immunosuppression of the Ducks

Dexamethasone (Dexamethasone sodium phosphate and Sodium methyl hydroxybenzoate, Coophavet, France) was used to stress ducks in this study. The respective groups of ducks were injected intramuscularly with the dexamethasone, following the protocol of Corrier et al. [7] modified as follows: the dosage was given at the rate of 2 mg per kilogram of body weight per day for 4 days continuously, then the ducks were rested for 2 days and the injections resumed at the same dosage for 2 more days.

### 2.4. Experimental Design

Sixty-four ducks were vaccinated with 1 mL of inactivated ND vaccine intramuscularly and 14 days later, they were bled from the brachial vein and sera prepared. They were later boosted with a single dose of 0.5 mL of the ND inactivated vaccine and bled 7 days later. All sera were tested for presence of Newcastle disease antibodies. Seven days after the booster dose, the ducks were divided into two groups, each with 32 birds, namely, low antibody level group (≤1 : 32) and medium antibody level group (≥1 : 64). Each group of 32 ducks was further subdivided into 4 minigroups, as follows: (i) immunosuppressed and challenged (1a, 2a), (ii) immunosuppressed only (1b, 2b), (iii) challenged only (1c, 2c), and (iv) not challenged nor immunosuppressed (1d, 2d). Another group (group 3) of 30 nonimmunized ducks were subdivided into 4 groups. Groups 3a and 3c had 12 ducks each while 3b and 3d had 3 birds each. Immunosuppression was done before respective groups were inoculated intranasally with 0.2 mL of undiluted amnioallantoic fluids of vNDV having a titer of 1 : 1024.

Five birds from each of the challenge groups and all the 3 ducks from each control group were sampled throughout the experimental period (28 days). The samples were taken on days 0, 1, 4, 8, 14, and 28-after inoculation (p.i.). Blood for serum was sampled each time from the five ducks in each challenge group, and the three ducks from each of the controls. Further, two ducks from each of the NDV challenged groups were killed serially and brain, kidney, lung, cecal tonsils, liver, and spleen collected separately from each bird. The tissues were processed for ND viral recovery using chicken embryo fibroblasts, while serum samples were tested for NDV-specific antibodies by hemagglutination inhibition (HI) test.  Table 1 shows the experimental design used.

### 2.5. Virus Isolation

Virus recovery from the tissues was carried out in primary chicken embryo fibroblasts as described by OIE [6].

### 2.6. Serology

Presence of NDV antibody was detected by hemagglutination inhibition test as described by OIE [6].

### 2.7. Statistical Analysis

Analysis of variance was performed using SAS software (SAS Institute Inc., Cary, NC, USA, 2002-2003) to determine the treatments' main effects and the interaction between time (days) and treatment, on various responses.

## 3. Results 

### 3.1. Serological Responses of Ducks under Different Treatments

**Figure 1:**
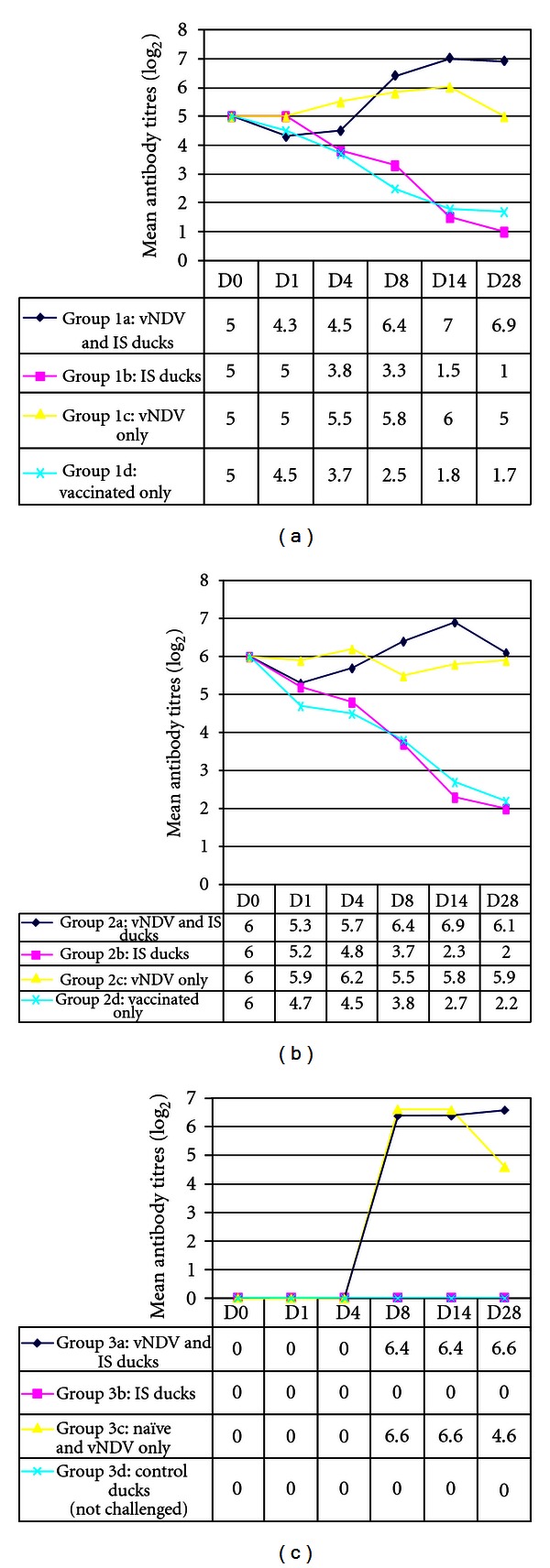
Mean antibody titre responses with respect to days (D) after challenge in immunosuppressed (IS) and nonimmunosuppressed, nonvaccinated ducks: (a) low antibody level (group 1); (b) medium antibody level (group 2); (c) nonimmunized (group 3).

Immunosuppressed virus-challenged ducks (group 1a) had low mean antibody levels (5.0) up to day 4 after-inoculation (p.i.) compared with day 0 (4.5). Thereafter, there was marked increase (from 4.5 to 7.0) in antibody titers up to 14 days p.i. After day 14 p.i., there was a slight decrease (6.9) in antibody levels up to 28 days p.i. although the levels were still higher than any period between day 0 and 8 p.i. The nonimmunosuppressed virus-challenged group (1c) had a moderate increase (5.0 to 6.0) in antibody levels from day 1 up to day 14 p.i., after which there was a decrease to day 0 level titers by day 28 p.i. The immunosuppressed group (1b) had marked decrease in antibody titers from day 1 to 4 and gradual decrease (5.0 to 3.8) up to day 28 p.i. (Figure 1(a)). 

The immunosuppressed virus-challenged group for the medium antibody level ducks (2a) had a gradual decline (6.0 to 5.7) in antibody titers up to day 4 followed by an increase in antibody titers (6.9) up to day 14 p.i. This was followed by a marked decrease (6.1) and by day 28 p.i. the antibody level was almost equal to the day 0 level titers. The nonimmunosuppressed virus-challenged group (2c) showed a slight decrease (from 6.0 to 5.9) in the antibody titer followed by a gradual decrease and then an increase up to the end of the experiment. From day 1 up to day 4 after-inoculation, the immunosuppressed, immunised noninfected (2b) group showed a more rapid decrease (6.0, 5.2, 4.8, 3.7, 2.3, and finally 2.0) in antibody levels as compared (6.0, 4.7, 4.5, 3.8, 2.7, and finally 2.2) to the nonimmunosuppressed controls (2d). In general, all the nonchallenged, but immunized control ducks showed decrease in antibody titers with time (Figure 1(b)). 

The immunosuppressed virus-challenged group (3a) had a gradual antibody response (from 0.0 to 6.5) up to the end of the experimental period. The nonimmunosuppressed virus-challenged group (3c) showed a massive increase (0.0 to 6.6) in antibody levels similar to immunosuppressed virus-challenged group 3a. The group 3c also had a marked decrease (from 6.6 to 4.6) in antibody titres after day 14 p.i. and by 28 days p.i., the titers were quite low. Negative control ducks (3b and d), sampled at the same time, were negative for NDV antibodies (Figure 1(c)). 

For days 4, 8, 14, and 28 p.i. antibody titres of the following groups were found to be significantly different (*P* < 0.05). When considering the antibody levels elicited in the different duck-groups, with respect to their initial antibody levels, both sets (low-antibody group and medium-antibody group), and both the immunosuppressed and non-immunosuppressed ducks showed higher responses in ducks that were challenged with virulent virus than in those that were not challenged. The difference was statistically significant (*P* < 0.05). All the control naive ducks (groups 3b and 3d) did not sero-convert. Immunosuppressed medium-antibody-level, challenged with NDV ducks (group 2a) versus immunosuppressed medium-antibody-level, nonchallenged (group 2b), was lowest in the latter group. Nonimmunosuppressed low antibody level, challenged with NDV ducks (group 2c) versus 2d, the latter group had lower levels of antibodies. In addition, antibody titres of group 1a versus 1d were significantly different (*P* < 0.05) on day 14, being lower in the latter. All the control naïve (groups 3b and 3d) birds did not seroconvert.

### 3.2. Isolation of Newcastle Disease Virus from Immunosuppressed and Nonimmunosuppressed Virus-Challenged Duck

**Figure 2:**
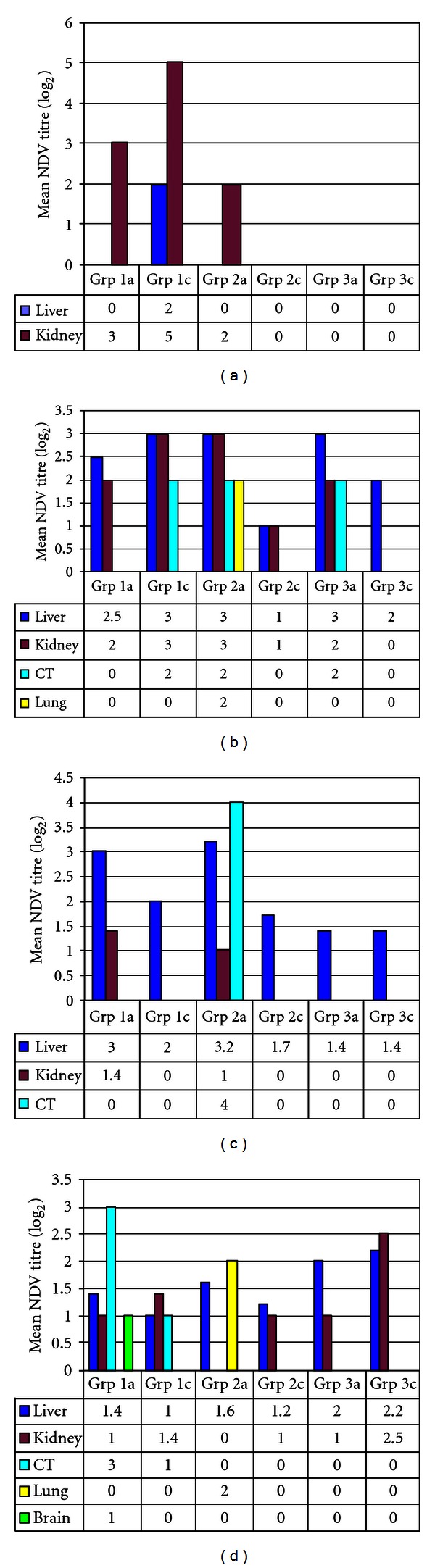
Newcastle disease viral titres in different tissues (liver, kidney, lung, brain, and caecal tonsils [CT]) of ducks in different treatment groups (Grp): (a) on day 4 after inoculation; (b) on day 8 after inoculation; (c) on day 14 after inoculation; (d) on day 28 after inoculation.

On day 1 after inoculation, NDV titers were recorded in liver tissues of group 1a (low-antibody-level group, immunosuppressed and challenged with vNDV) ducks only. On day 4 p.i., high titres of the NDV were recorded in the kidneys (Figure 2(a)). On day 8 p.i., NDV was isolated in the liver, kidneys, caecal tonsils, and lungs of all treatment groups (Figure 2(b)). The highest NDV titers were recorded in the liver and kidney tissues of immunosuppressed medium (2a) and nonimmune (3a) challenged ducks and nonimmunosuppressed, low-antibody-level challenged ducks (1c). No NDV was isolated by day 14 p.i. in the brain and spleen from any of the groups. 

On day 14 p.i. high NDV titres were recorded from the liver tissues of ducks in all treatment groups. However, titres were recorded in the cecal tonsils only in group 2a on day 14 p.i. and in groups 1a and 1c at day 28 p.i. (Figures 2(c) and 2(d)). Other organs that were positive for NDV were kidneys and cecal tonsils. In addition, the immunosuppressed ducks of groups 1a (low-antibody-level group, immunosuppressed and challenged with vNDV) and 2a (medium-antibody-level group, immunosuppressed and challenged with vNDV) yielded the highest NDV titres as compared to other treatment groups. Newcastle disease virus was recovered from the brain and lung on day 28 p.i from immunosuppressed ducks only (Figure 2(d)).

## 4. Discussion 

There have been comparatively few sequential virological studies on the pathogenesis of ND in ducks and the reported studies involved only fully susceptible chickens [8, 9]. Reports in other studies have documented frequent isolation of virulent NDV from captive caged birds, healthy wild birds, and village chickens [10–12]. In some cases, the ducks expressed clinical ND as a result of confinement that was understood to have induced stress [13]. Our findings indicate that NDV carrier status in nonnatural hosts such as ducks would possibly, under stress, recrudescence virulent virus from sequestered sites in the kidney, liver, and caecal tonsils, leading to virus release in faecal and respiratory exudates. The excreted virus would set up an infectious index case in chickens, thus maintaining NDV endemicity.

Immunosuppression induced by injection of dexamethasone in the three treatments influenced the pattern of antibody response and the NDV recovery rate. The immunosuppressed ducks that had low and medium antibody level showed a decrease in antibody titers up to day 4 after challenge with NDV. The nonimmunosuppressed virus-challenged ducks of low-to-medium antibody level had an increase in antibody titres up to day 14 p.i. The nonimmunized ducks manifested increased antibody titres after day 4 p.i. and had a massive increase in antibody levels as compared to immunosuppressed challenged group. In the present study, the number of immunosuppressed ducks that yielded the ND virus was higher compared to the nonimmunosuppressed. The prechallenge antibody titers may therefore play a significant role in shedding off the virus as well as clinical manifestation of the disease. Gessani et al. [14] noted that a few hours of treatment with low concentrations of synthetic glucocorticoid (analogue dexamethasone) are sufficient to inhibit the synthesis of interferon, a virus inhibitor. This may, in part, explain the observation that treatment with glucocorticoids increased virus yield and lethality in mice infected with Coxsackie virus [15]. Our present study using ducks concurs with those of Asdell and Hanson [16] who showed that prior treatment of chickens with dexamethasone lead to massive ND virus multiplication. 

There was significant difference in geometric mean antibody titers between the immunosuppressed ducks of group 1a and nonimmunosuppressed counterparts (group 1d) and also between immunosuppressed ducks of group 3a and nonimmunosuppressed group 3d. This means that whereas dexamethasone seems to have an effect on immune system of NDV-infected ducks, the prechallenge titres also play a major role in the immune response of immunosuppressed birds in that immunosuppression of ducks with high viral titers allows virus multiplication making ducks better carriers. The effect of immunosuppression with dexamethasone in ducks appears to be the same as that induced by aflatoxin in chicks with NDV infections [17]. Chickens fed on aflatoxin produced lower antibody levels when compared to the non-aflatoxin-treated ones [17]. 

The nonchallenged preimmunized ducks had a progressive decrease in antibody levels suggesting that if they were to be exposed to the virus, they could come down with the ND or if the antibody titers were within the protective levels (2^4^ to 2^7^), they might not develop clinical disease but instead may remain as virus carriers. The fact that the ducks in these experiments had high levels of antibodies may not necessarily prevent subclinical infection and excretion of virulent virus as supported by other studies elsewhere [18]. 

Based on these results, it is possible to assume that immunosuppressed ducks carrying NDV are more likely to shed virus under stress than nonimmunosuppressed ducks. Furthermore, the ducks showed progressively declining antibodies titres to very low levels making them more susceptible to infection by challenge ND virus, thus leading to clinical disease and virus excretion. The excreted virus would contaminate the birds' environment and be transferred to susceptible chickens and other birds. Thus, immunosuppression seems to have epidemiologically significant effects on NDV carrier ducks having varying antibody titer levels.

## Figures and Tables

**Table 1:** Groups of ducks used to evaluate the effect of immunosuppression on persistence of Newcastle disease virus under different treatments.

Antibody grouping of ducks	Group code of ducks	Number of ducks	Treatments		
			Dexamethasone	vNDV	Vaccination
Low antibody level	1a	13	+	+	+
	1b	3	+	**−**	+
	1c	13	−	+	+
	1d	3	**−**	**−**	+

Medium antibody level	2a	13	+	+	+
	2b	3	+	**−**	+
	2c	13	**−**	**+**	+
	2d	3	−	−	+

Nonimmunized	3a	12	+	+	−
	3b	3	**+**	−	−
	3c	12	−	+	−
	3d	3	−	−	−

+: respective treatment administered; −: no treatment; vNDV: velogenic Newcastle disease virus; Groups tabulated as “a”: immunosuppressed and challenged with vNDV disease virus; Groups tabulated as “b”: immunosuppressed but not challenged; Groups tabulated as “c”: not immunosuppressed but challenged with vNDV; Groups tabulated as “d” are controls for group c: not immunosuppressed nor challenged; Group 3: comprises unvaccinated controls for the 4 test groups.
